# Disentangling non-specific and specific transgenerational immune priming components in host–parasite interactions

**DOI:** 10.1098/rspb.2019.2386

**Published:** 2020-02-12

**Authors:** Frida Ben-Ami, Christian Orlic, Roland R. Regoes

**Affiliations:** 1 School of Zoology, George S. Wise Faculty of Life Sciences, Tel Aviv University, Tel Aviv 6997801, Israel; 2 Zoologisches Institut, Evolutionsbiologie, Universität Basel, Vesalgasse 1, Basel 4051, Switzerland; 3 Institute of Integrative Biology, ETH Zurich, Zurich 8092, Switzerland

**Keywords:** immune priming, trained immunity, host heterogeneity, *Daphnia magna*, *Pasteuria ramosa*, mathematical modelling

## Abstract

Exposure to a pathogen primes many organisms to respond faster or more efficiently to subsequent exposures. Such priming can be non-specific or specific, and has been found to extend across generations. Disentangling and quantifying specific and non-specific effects is essential for understanding the genetic epidemiology of a system. By combining a large infection experiment and mathematical modelling, we disentangle different transgenerational effects in the crustacean model *Daphnia magna* exposed to different strains of the bacterial parasite *Pasteuria ramosa*. In the experiment, we exposed hosts to a high dose of one of three parasite strains, and subsequently challenged their offspring with multiple doses of the same (homologous) or a different (heterologous) strain. We find that exposure of *Daphnia* to *Pasteuria* decreases the susceptibility of their offspring by approximately 50%. This transgenerational protection is not larger for homologous than for heterologous parasite challenges. Methodologically, our work represents an important contribution not only to the analysis of immune priming in ecological systems but also to the experimental assessment of vaccines. We present, for the first time, an inference framework to investigate specific and non-specific effects of immune priming on the susceptibility distribution of hosts—effects that are central to understanding immunity and the effect of vaccines.

## 1. Introduction

Transgenerational effects occur when the phenotype of the parent affects the phenotype of its offspring in addition to the direct effects of the genes contributed by the parent [1–3]. These effects are ubiquitous in nature and have been documented in a wide range of traits and taxa [4–8].

Among the most widely studied transgenerational effects are those involving the transfer of immunity or increased parasite resistance from parents to offspring, commonly found in vertebrates [9,10], but also in invertebrates [11–13]. The latter is particularly intriguing, because until the beginning of this millennium the innate immune system of invertebrates was thought to be capable of only non-specific responses that were unaffected by previous exposures to parasites (e.g. [14]). The potential of innate immune systems to specifically remember previous exposures to pathogens was first supported by phenomenological evidence (reviewed in [15–17]). In particular, it has been shown that invertebrate hosts can be primed against specific parasite species and strains, and the priming effects can extend across life stages and generations [18–22]. There is also growing evidence that the innate immune system of invertebrates shares several homologies with that of vertebrates [16,23,24], although it has been argued that immune memory in invertebrates may be mediated by yet unidentified mechanisms that will not be found by looking for homologies [15,25]. More recently, the interest in potential innate immune memory has been revived, and new molecular mechanisms are being elucidated in invertebrates [26–28], and even in vertebrates [29].

Immune priming can be non-specific or specific. Specificity here defines the degree to which a primed immune response is able to discriminate among different parasite strains, species, or taxa (e.g. Gram-positive bacteria; [12]). While non-specific immune priming is important for eliciting a general response against a variety of parasites, specific immune priming can provide a targeted, and often more effective and long-lasting protection against reinfections. It is thus crucial to disentangle non-specific from specific immune priming in order to understand which of the two is responsible for an observed immune response.

Studies of transgenerational effects on disease typically subject the parental environment to food stress, for example, shortage of food or food of lower quality [30,31], crowding [32] or challenge them with live, weakened, or heat-killed parasites [33–35]. Thereafter, a variety of traits of the offspring generation are recorded, such as susceptibility to parasites and offspring fecundity, resistance, immunity, and mortality [36–38]. The vast majority of studies on transgenerational effects focused on non-specific immune priming [34,35,39–42]. Only a handful of studies involving invertebrates found evidence for specific transgenerational immune priming. For example, in a serial passage experiment, in which populations of the flour beetle *Tribolium castaneum* were subjected to a regime of challenge with heat-killed and subsequent infection with live *Bacillus thuringiensis* for 11 generations, Khan *et al.* [43] found evidence for the evolution of strain-specific immune priming in the beetles. In another recent study, Norouzitallab *et al.* [44] showed the occurrence of specific immune memory in the brine shrimp *Artemia franciscana*, manifested by increased resistance of the progeny of Vibrio-exposed ancestors towards a homologous bacterial strain when compared with a heterologous strain. Little *et al.* [45] obtained similar results in the crustacean *D. magna* by exposing mothers to one strain of *Pasteuria ramosa* and testing their offspring’s fertility following exposure to the same and a different strain.

Because specific immune priming can play an important role in host–parasite interactions at the population level, we combined an experiment with mathematical modelling to disentangle transgenerational effects of non-specific and specific immune priming in *D. magna* and its bacterial parasite *P. ramosa*. We used three isolates of *P. ramosa* to prime mother *Daphnia*, and exposed their offspring to all three isolates in a 3 × 3 factorial experiment. This resulted in nine treatment arms: three arms with homologous challenges, and six arms with heterologous challenges.

Instead of exposing host individuals to a single challenge dose of the pathogen, as is done in most studies, we used seven challenge doses ranging over more than five orders of magnitude. We chose multiple challenge doses because this allows us to study not just the average susceptibility but the entire distribution of susceptibilities in the host population under investigation. In particular, this approach can identify if priming affects each host individual uniformly, or if the host response to priming differs across hosts.

The main questions we address are if the susceptibility distribution is affected by priming, and if these potential effects differ for homologous or heterologous challenges. Hereby, we do not only consider priming effects on the mean susceptibility to challenge, but also effects on the variance of susceptibilities across hosts.

## 2. Results

### (a) Dose dependence of infection rates

To determine the existence and extent of these various forms of transgenerational immune priming, we conducted experiments with *D. magna* and three isolates of its parasite *P. ramosa*, P1, P2, and P5. We first exposed genetically identical *Daphnia* to a high dose of one of the three parasite isolates. The exposure lasted 7 days, after which the medium was replaced by parasite-free medium. As a control, a subset of the *Daphnia* were not exposed to any parasite strain. All unexposed control animals remained uninfected throughout the experiment. Overall, this resulted in four treatment groups.

**Figure 1.**
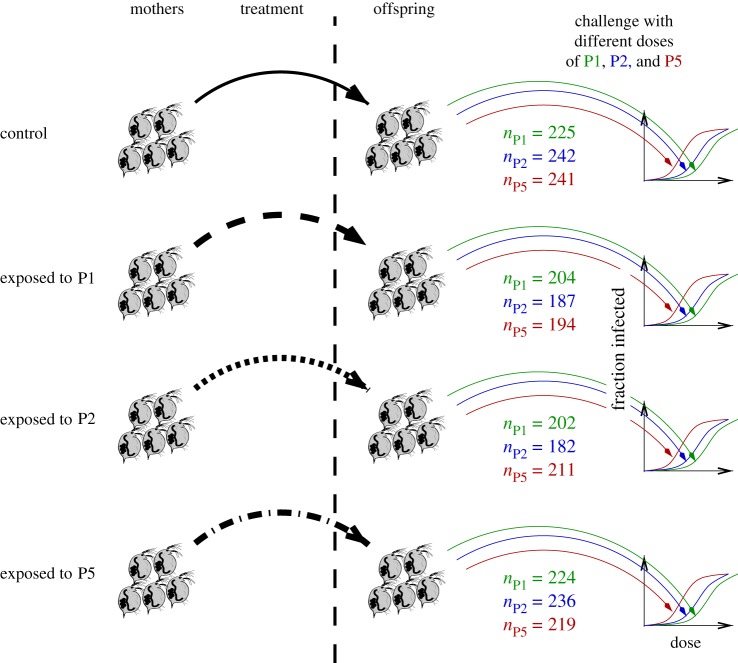
Design of our experiment. Mother *Daphnia* were exposed to three different strains of *Pasteuria ramosa*, P1, P2, or P5. A control cohort of mother *Daphnia* was not exposed. The offspring of these mothers were then exposed to seven different challenge doses of P1, P2, or P5. The sample sizes for each group are indicated on the diagram. They amount to approximately 20–30 individuals per strain and challenge dose. In total, we used 2567 individuals. (Online version in colour.)

The exposed and control *Daphnia* subsequently produced offspring. All offspring were produced after the exposure of the mothers. We excluded offspring from mothers that did not become infected during the experiment (see electronic supplementary material). We then challenged the offspring individuals from the four treatment groups with seven different doses of each of the three parasite strains (figure 1). Lastly, we assessed the infection status of *Daphnia* offspring 39 days after exposure (on day 44).

**Figure 2.**
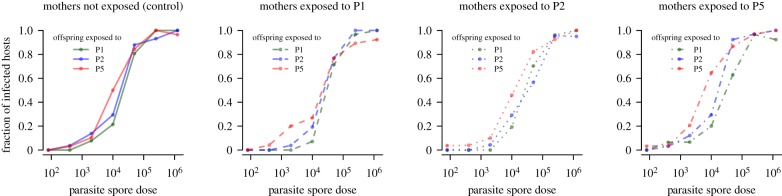
Fraction of infected hosts versus parasite challenge dose for each maternal treatment group. The colour and line type scheme is chosen in concordance with the experimental design schematic shown in figure 1. For a figure showing these data by offspring parasite, see electronic supplementary material, figure S2. (Online version in colour.)

The main readout from the experiment is the fraction of infected *Daphnia* as a function of the exposure dose in the homologous and heterologous challenge groups, as well as in the control group (figure 2). Since these data are the result of potentially competing influences of specific and non-specific transgenerational immune priming, a formal method was required to disentangle the effects of maternal exposure on offspring susceptibility.

### (b) Modelling framework

To analyse these data, we extended a mathematical framework we had developed previously. The original framework allowed us to estimate the average infection probability and its inter-individual variance [30,46,47]. The inspiration for our previous work came from frailty mixing models in mathematical epidemiology [48–50], but the approaches are also used in microbial risk assessment [51,52]. The original framework, however, was conceived to analyse infection experiments involving only a single parasite strain and to contrast the susceptibilities of *Daphnia* whose mothers had or had not been exposed to this parasite strain [30,47].

To be able to address the question of non-specific versus specific immune priming in our infection experiment, we extended this framework by incorporating parameters that capture many of the conceivable ways of how exposure of the mothers to a specific parasite strain can alter the susceptibility of the offspring to infection (see electronic supplementary material). This is needed to analyse the results of our fully factorial experiment, in which both the mother and offspring generation were exposed to three parasite strains. In our modelling framework, the baseline susceptibilities of control *Daphnia* to each of these three parameters are denoted by *b*_01_, *b*_02_, and *b*_05_. Hereby, the indices 01, 02, and 05 denote the three control groups of *Daphnia*. The first index ‘0’ signifies that the mothers of the *Daphnia* in these groups were not exposed to a parasite. The second index, ‘1’, ‘2’, or ‘5’, denotes the isolate to which the offspring was exposed, P1, P2, and P5, respectively.

In our extended framework, we separated the potential priming-induced alterations of susceptibility into a heterologous and a homologous component. For example, if the mother *Daphnia* was exposed to P1, its offspring may be less susceptible to challenge with any parasite strain. This would constitute non-specific immune priming against heterologous challenge, and is captured by the model parameter *r* (see electronic supplementary material). Alternatively, maternal exposure to P1 could reduce susceptibility of offspring to P1 specifically, that is, it could partially protect the offspring against homologous challenge. This specific memory effect is captured by the model parameter *m* (see electronic supplementary material). Our modelling framework also allows for non-specific and specific alterations of the variance of the susceptibility distribution. These effects are captured by the parameters *ρ* and *μ*, respectively (see electronic supplementary material).

Our modelling and inference framework has clear advantages over the more commonly applied generalized linear modelling approach. These are detailed in the electronic supplementary material to this paper.

### (c) Baseline susceptibility and heterogeneity estimates

First, we estimated the average susceptibility to each parasite strain *b*_0*j*_ and its variance *v*_0*j*_ from the infection data of the control group. The baseline susceptibility estimates are an important reference point against which we later tested for non-specific and specific immune priming effects. For P1, P2, and P5, respectively, we obtain *b*_01_ = 8.73 × 10^−5^, *b*_02_ = 1.68 × 10^−4^, and *b*_05_ = 2.49 × 10^−4^ as the average susceptibilities, and *v*_01_ = 10^−9^, *v*_02_ = 0.73, and *v*_05_ = 0.91 as the susceptibility variances. Figure S4 in the electronic supplementary material shows the likelihoods for the control data. Figure S3A in the electronic supplementary material shows the fits to the control data.

To study if there is non-specific or specific transgenerational immune priming, we adopted a model selection scheme. We constructed models with increasing complexity, the simplest of which assumes no immune priming effects, and serves as a null model in our analysis. Table 1 lists and defines the models we considered. By fitting the models in order of increasing complexity to our experimental data and comparing the quality of the fits statistically, we test for the existence of non-specific and specific immune priming effects (see electronic supplementary material).

**Table 1.** Model variants considered in the model selection scheme. The highlighted *r* − *m*_*i*_ model has the strongest statistical support.

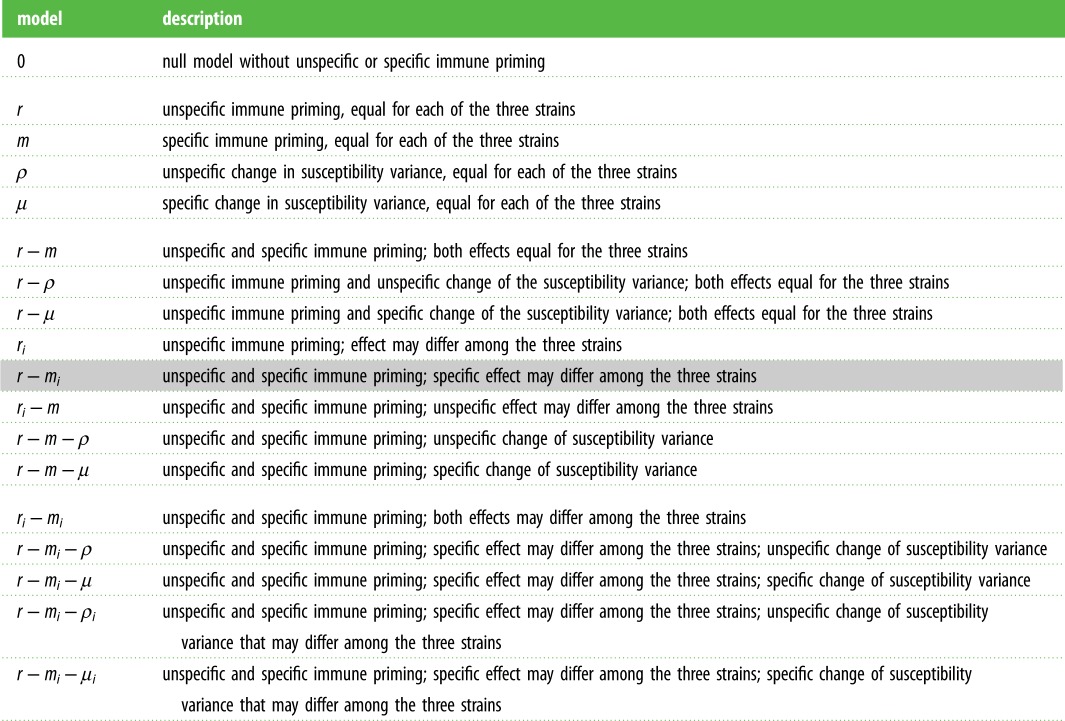

### (d) Evidence for non-specific immune priming

**Figure 3.**
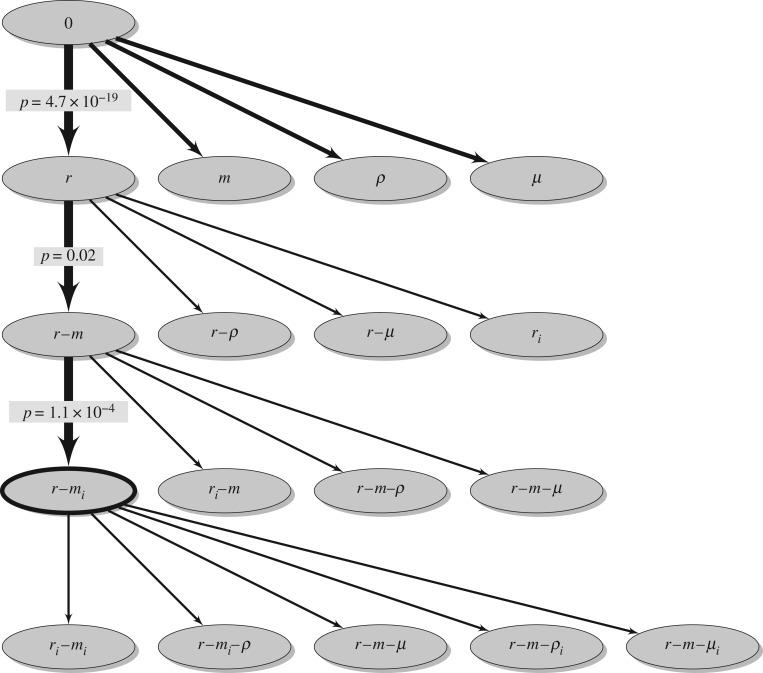
Model selection scheme. The thick arrows denote statistically significant model improvements. Statistical significance was determined by a likelihood ratio test between two models. The *p*-values of these tests are shown. The thick ellipse circles the most complex model with statistical support.

We tested four models that are one step more complex than the null model: *r*, *m*, *ρ*, and *μ* (table 1 and figure 3). These model extensions test for the existence of overall immune priming effects of maternal exposure on the average susceptibility or its variation. But this potential effect is assumed not to differ between the parasite strains P1, P2, and P5.

**Figure 4.**
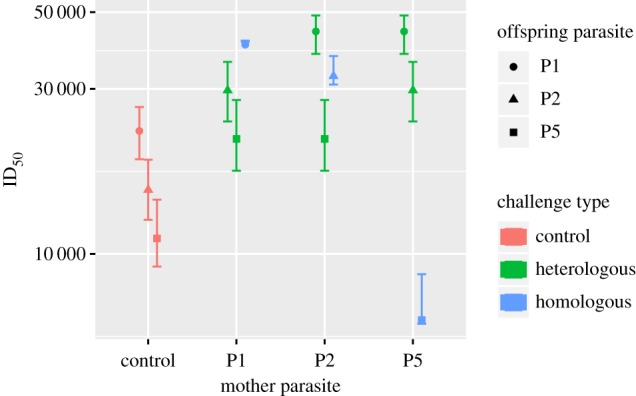
ID_50_ by maternal treatment group. Vertical bars show the standard error of the ID_50_ estimates that were calculated by bootstrap (see electronic supplementary material). (Online version in colour.)

We fitted all four model extensions maximizing the likelihood function described in the electronic supplementary material. While all of these model extensions fit significantly better than the null model, the largest improvement in fit arises from the *r* model that describes a non-specific, cross-strain immune priming effect (likelihood ratio test: *p* = 4.7 × 10^−19^). We estimate an effect *r* = 0.43. This means that maternal exposure, irrespective of the specific parasite strain, reduces the average susceptibility of the offspring to any strain by 43%. This reduction translates into an approximately twofold increase of the ID_50_ (figure 4).

### (e) No evidence for specific immune priming

Because the *r*-model resulted in the largest improvement of model fit we used it as a baseline for the subsequent analysis. We considered four models that are one step more complex than the *r*-model (table 1). Biologically, the most relevant of these are the *r* − *m*-model and the *r*_*i*_-model. The *r* − *m*-model allows for specific immune priming in addition to the non-specific effect already described in the *r*-model. This specific effect is captured by the parameter *m* that denotes the fraction by which the susceptibility to homologous challenge is reduced. The *r*_*i*_-model extends the *r*-model by accommodating potential differences between the non-specific immune priming effects of each parasite strain.

Of the four conceivable models, only the *r* − *m*-model improves the fit significantly (likelihood ratio test: *p* = 0.02; see also figure 3). Thus, we have evidence for a specific, transgenerational memory of parasite strains. However, the parameter *m* in this model, which describes how well the maternal parasite strain is remembered, is negative: *m* = −0.40. This can be interpreted as specific facilitation of infection, rather than specific protection, and is thus the opposite of immune priming.

### (f) Maternal exposure to P5 facilitates infection with P5

The *r* − *m*-model can be extended in various ways (table 1 and figure 3). The most relevant extensions are the *r* − *m*_*i*_-model and the *r*_*i*_ − *m*-model. The *r* − *m*_*i*_-model assumes that maternal exposure to any of the three parasite isolates reduces the susceptibility of the offspring non-specifically by the same fraction *r*. The model further assumes three specific effects, measured by *m*_*i*_, describing how maternal exposure to each parasite isolate reduces the susceptibility of the offspring to homologous challenge with the same parasite isolate. The *r*_*i*_ − *m*-model, by contrast, assumes that the non-specific reduction of the susceptibility of offspring differs for each of the parasite isolates, to which the mothers were exposed. The specific effect, on the other hand, is assumed to be the same for each parasite isolate, that is, the offspring’s susceptibility to homologous challenge is assumed to be reduced by the same fraction *m* for each parasite isolate.

Of all the extensions, we considered, however, only the *r* − *m*_*i*_-model improves the fit significantly (likelihood ratio test: *p* = 1.1 × 10^−4^; see also figure 3). The fit of this model is not improved by any further model extensions (figure 3, bottom row). Hence, the *r* − *m*_*i*_-model represents the model complex enough to capture all aspects related to non-specific and specific immune priming in the data without over-fitting them. Figure S3B in the electronic supplementary material shows the fits of the best model (*r* − *m*_*i*_ model) to each group of the data.

The non-specific effect of maternal exposure in the *r* − *m*_*i*_-model is estimated as *r* = 0.48 with a 95% confidence interval between 0.39 and 0.55. This means that maternal exposure to a parasite isolate reduces the susceptibility of the offspring to any of the three parasite isolates by 48%. The three parameters describing specific immune priming are estimated as *m*_1_ = −0.054 (−0.56, 0.29), *m*_2_ = 0.10 (−0.50, 0.46), and *m*_5_ = −2.16 (−4.07, −0.97). The numbers in parentheses give the 95% confidence intervals. Importantly, only *m*_5_ is estimated to be significantly different from 0, and is negative. This means that exposing mothers to P5 facilitates infection with P5. This surprising effect is also reflected in the low ID_50_ estimate for this treatment group (see the lowest point in figure 4).

## 3. Discussion

In the present study, we exposed *D. magna* mothers to three strains of the bacterial parasite *P. ramosa*, and then challenged the offspring homologously or heterologously. Our aim was to determine if there are transgenerational effects of parasite exposure on the distribution of host susceptibility to infection, and if these are driven by non-specific or specific immune priming. We found strong evidence of non-specific, cross-strain immune priming, which decreases the susceptibility of host offspring to infection by approximately 50%. We found no evidence of specific immune priming that reduces susceptibility to infection with the same strain, with which the mother had been challenged (homologous exposure), when compared with the susceptibility to infection with other strains (heterologous exposure). However, we found that maternal exposure to one particular parasite isolate (P5) facilitates, rather than prevents, offspring infection with this parasite.

A previous study on the same host-parasite system found evidence for strain-specific immune priming [45]. We, by contrast, did not find evidence of specific immune priming fine enough to distinguish among different strains of *Pasteuria*. One important difference between the two studies lies in the trait considered to be affected by maternal parasite exposure: we considered susceptibility to challenge and its variation across individuals in the population, while Little *et al.* [45] focused on offspring fecundity. Furthermore, approximately 1000-fold differences in sensitivities between the two strains used by [45] might indicate a confounding dose effect. Alternatively, specific immune priming might be driven by genotype-by-genotype (GxG) interactions, which are well documented in this system [53], insofar the specific host–parasite combinations would be more likely than others to exhibit specific immune priming. The conflict between our study and that of [45] should not be conflated with the more fundamental criticism voiced against studies of invertebrate immunity [54,55]. As many studies of invertebrate immunity, we did not investigate the molecular basis of the effects we found. Demonstrating increased offspring survival or resistance to parasites following prior parental exposure does not necessarily require the involvement of immunity [56]. But, by relying on the formal concepts and the experimental design principles of population biology, we succeed in elucidating the phenomenological effects of previous exposure on host susceptibility to a yet unsurpassed level of detail.

The mean susceptibility of *Daphnia* to isolate P1 and its variance were significantly lower in comparison with P2 and P5. These results are consistent with earlier studies of P1 and two other *Pasteuria* isolates, P3 and P4, which showed that P1 had the lowest infectivity [47]. Moreover, in a variety of mixed infection scenarios, P1 was found to be more virulent but produced fewer spores than isolates P3/P4 and clone C1 (obtained from isolate P5, [47,57]). If virulent strains produce fewer transmission stages, this could influence the generation of specific immune priming and transgenerational memory.

Our results are consistent with previous studies of transgenerational effects of *Pasteuria* exposure on *Daphnia* susceptibility [30,47]. In those studies, we exposed *Daphnia* to only one *Pasteuria* isolate (P5). Thus, we could not test for specific immune priming. The parameter estimates for this isolate we obtained here however, are inconsistent with those from our previous study [30]. The mean susceptibility of the control group was almost fourfold lower, whereas mean susceptibility of the exposed group was less than twofold lower than previously. Consequently, in the present study, mean susceptibility of the control group was 33% higher than that of the exposed group, whereas in the previous study, it was 38% lower. While the susceptibility variance of the control group between our two studies are consistent, in the present study exposure to isolate P5 did not lead to the significant increase in the susceptibility variance we found previously.

Since the comparison between the present study and the previous one [30] was unplanned, it is not surprising to find differences between the two studies. Nevertheless, it is important to carefully consider what factors could have led to such a divergence between the studies in the mean and variance of offspring susceptibility in the treatments with control and P5-exposed mothers. First, environmental conditions such as food availability and host density can influence maternal effects [31,32,58]. The duration of exposure could also influence susceptibility [59,60]. However, daily food levels of control and exposed treatments and the duration of exposure of exposed treatments in this study were very similar to those in the previous one [30]. Second, phenotypic heterogeneity in host susceptibility to environmental and physiological factors, such as molecular differences in immune response [61] and within-clone variation in life-history traits (e.g. differences in size at birth; [62]), could also influence the mean and variance of offspring susceptibility. Such heterogeneity would, however, not explain why maternal exposure to parasite isolates P1 or P2 did not facilitate offspring infections as it did for P5. Lastly, the genetic composition of parasite isolate P5 might have changed across studies. Isolates are parasite samples from infected hosts that may contain multiple genotypes [63]. They are a naturally occurring feature of the *Daphnia-Pasteuria* host–parasite system. In the laboratory, isolates are propagated through experimental hosts, to obtain enough spore-carrying cadavers to produce sufficient amounts of spore suspensions. Thus, it might be that over time, some genotypes within the P5 isolate have changed in frequency.

Transgenerational immune priming has been described in a variety of taxa, including insects and crustaceans (reviewed in [56]). Our work adds to the growing literature on transgenerational immune priming in invertebrates. While most studies could not disentangle non-specific from specific immune priming by design because the mothers and the offspring were exposed to the same parasite strain, there is mounting evidence of specific immune priming in invertebrates [33,43–45]. In this study, we present the most extensive dataset and analysis of transgenerational specific immune priming in invertebrates to date. While we find clear evidence for non-specific immune priming across generations, our results on specific immune priming are basically negative. Our evidence for a specific priming effect applies only to one of the three *Pasteria ramosa* isolates (P5) and goes into the ‘wrong’ direction of facilitation, rather than protection. Our study thus shows the limits of specificity of immune priming in *Daphnia*. According to our findings, *Daphnia* do not inherit a memory of the specific *P. ramosa* isolate to which they were exposed. Our study emphasizes that there are limits of specificity, even in systems where specific immune priming effects have been established. Determining these limits can contribute to identifying the often elusive molecular mechanisms that confer specific priming effects in invertebrates.

The fact that transgenerational immune priming is widespread suggests that this trait has adaptive value. Two evolutionary hypotheses have been proposed [56]. First, the transfer of immunity to offspring may protect it when it is not yet able to mount its own effective responses. This hypothesis essentially focuses on trade-offs between different life stages (reviewed in [64]). Because we exposed the offspring generation early in life, this hypothesis could, at least in part, be behind the priming effect we have established. Second, if the maternal pathogen environment resembles that of the offspring, immune priming is evolutionary advantageous. Hereby, the exact degree of specificity that is most adaptive depends on how likely it is that mother and offspring are exposed to the same type or strain of pathogen [65,66]. The non-specific priming effects we have found could have evolved in response to persistent pathogen pressure across generations. The fact that we could not find evidence for specific immune priming is consistent with an evolutionary scenario, in which subsequent generations face pressure from various types of pathogens rather, than the same strain of a pathogen, such as *P. ramosa*.

Formally, our work represents an important contribution not only to the analysis of immune priming effects in ecological systems but also to the experimental and epidemiological assessment of vaccines. In the epidemiological setting, frailty models have been used to infer the distribution of susceptibilities and vaccine effects [50]. Most importantly, this line of research gave rise to a more refined perspective on vaccine effects delineated by the two extreme scenarios of leaky and all-or-none effects [48–50,67,68]. A leaky vaccine effect describes a scenario in which the susceptibility of each vaccinated individual is reduced by the same factor. The all-or-none scenario, on the other hand, reflects a vaccine that is 100% effective in a subpopulation of vaccinees, and completely ineffective in the remaining population. These refined concepts of vaccine efficacy have been successfully used to infer the effect of vaccines in the epidemiological setting [49]. In experimental settings, in which the challenge dose and schedule can be better controlled, repeated low-dose challenges or challenges with multiple doses have been used to determine vaccine effects beyond the average reduction of susceptibility [69,70]. Also, the potentially immunizing effect of challenges in repeated schedules has been investigated [71]. However, an extension of these frailty modelling approaches to investigate specific and non-specific effects of immune priming has not been available to date. In this study, we provide such an extension. Furthermore, in our specific host-parasite system the lack of evidence for priming effects on the variance parameters of the susceptibility distributions strongly suggest a predominantly leaky mode of action of the non-specific transgenerational priming on susceptibility.

More generic statistical approaches to analysing the type of experiments we conducted, such as generalized linear models, have a number of shortcomings (see electronic supplementary material). Most importantly, they need to be tweaked to distinguish between homologous and heterologous challenges. Moreover, they do not allow to account for heterogeneity in susceptibility. These aspects, however, are central to understanding immunity and the effect of vaccines because immune memory and vaccines are typically specific to certain pathogen strains. Because vaccines aim to provide specific protection against certain pathogen strains, our inference framework will, therefore, be of use much beyond the example of *D. magna* and their parasites that we presented here.

## 4. Material and methods

### (a) Study organisms

*Daphnia magna* Straus is a cyclical parthenogenetic zooplankton, found in a variety of freshwater habitats, such as ponds and rain pools. In nature, many populations are found to be infected by numerous bacterial, microsporidial, and fungal parasites [72–74]. In the laboratory, clonal lines can be kept for many generations, allowing the exclusion of genetic effects experimentally. One of the most common obligate endoparasites of *D. magna* is the bacterium *P. ramosa* Metchnikoff 1888. This bacterial parasite castrates its host and has a strictly horizontal transmission strategy, by releasing spores from the cadaver of infected *Daphnia* that are ingested by susceptible *Daphnia* [75,76]. The castration, however, is not immediate and hosts can exhibit a burst of reproduction prior to death [76–78].

### (b) Experimental design

Our experimental design is summarized in figure 1. In brief, we initially either exposed *Daphnia* mothers to one of three *P. ramosa* isolates (P1: Gaarzerfeld, Germany, 1997; P2: Kaimes, England, 2002; P5: Moscow, Russia), or left them unexposed as controls. These isolates are parasite samples from infected hosts that may contain multiple genotypes [63]. Isolates are a naturally occurring feature of the *Daphnia-Pasteuria* host–parasite system, and are thus relevant to evolutionary processes in natural populations [76]. Despite the potential genetic heterogeneity of the parasite isolates, specific, heritable interactions with the host have been observed [79].

We exposed the mothers in the exposed groups to the parasite for 7 days. After this time the medium was replaced. The generation of offspring by the mothers occurred after the exposure. Thus, the risk of early exposure of the offspring to the parasite is minimal.

We subsequently collected the offspring of the mothers. The number of offspring per mother ranged from one to seven with a median of two. In the exposed groups, we only included the offspring of mothers that became infected upon exposure (by collecting all offspring and using them in the second-generation experiment, and later discarding from the analysis those hosts whose mothers had not been infected). This was done to ensure that the exposure treatment was as homogeneous as possible. We exposed the offspring to different doses of all three *Pasteuria* isolates, thereby creating one homologous and two heterologous groups per maternal treatment group and dose. We used a single laboratory-maintained *D. magna* clone (HO2 from Hungary) in order to exclude genetic variation among hosts apart from mutations. Isolate P5 was used in a previous study of maternal effects of *D. magna* [30].

For the mothers’ generation, we placed 4-day-old juveniles individually in 100 ml jars with 20 ml of artificial medium (ADaM; [80]), and on day 5 all individuals in the exposed treatments were challenged with 50 000 spores of the respective *P. ramosa* isolate. We fed the animals 1 × 10^6^ algae cells of *Scenedesmus gracilis* per *Daphnia* per day. On day 12, we replaced the medium of all animals with 100 ml of fresh medium, and thereafter changed the medium every week. We increased the food levels on days 6, 9, 11, and 13 to 2 × 10^6^, 2.5 × 10^6^, 3 × 10^6^, and 8 × 10^6^ algae cells per individual per day, respectively, to accommodate the growing food demand.

For the second generation, we collected offspring daily from the mothers and, at an age of 4 days, offspring were singly placed in 100 ml jars with 20 ml of medium. We assigned the offspring of each mother group randomly to one of seven dose levels (80, 400, 2000, 10 000, 50 000, 250 000, and 1 250 000 spores/animal) or to a control group. On day 5, we exposed all individuals to its respective parasite strain/dose combination, and after a week, the medium of all animals was replaced with 100 ml of fresh medium. Thereafter, we applied medium replacement and feeding schedules identical to those that we had applied to the mothers. We kept both, mothers and offspring, at 20 ± 0.5°C, and set the light:dark cycle ratio to 16 : 8 h. We distributed the jars from all treatment groups randomly across the shelves in a controlled climate room, and rearranged them frequently to prevent position effects.

When offspring individuals died, we recorded the day of their death. The main cause of death of offspring was injuries inflicted when we separated them from their mothers. We assessed individuals that died for infection only if their death occurred more than 16 days after their birth because infection cannot be reliably determined earlier. Animals that died earlier were excluded from the analysis. We ended the experiment on day 44, and scored all animals by eye for infection by examining the colour of infected animals, which lose their typical transparency and turn brownish-red, and also lack eggs. In cases in which we could not unambiguously determine the infection status by eye, we dissected the animal to corroborate infection using a phase-contrast microscope (300–600X).

### (c) Mathematical modelling

While the mathematical modelling and inference framework constitutes a key outcome of our research, and although it is essential to our study, we moved the comprehensive description of the modelling into the electronic supplementary material to comply with the page limit of the Proceedings of the Royal Society B.

## Supplementary Material

Supplementary Text and Figures

Reviewer comments

## Supplementary Material

Experimental Data

## Data accessibility

We submitted the experimental data and R-code as electronic supplementary material.

## References

[1] Räsänen K, Kruuk L 2007 Maternal effects and evolution at ecological time-scales. Funct. Ecol. 21, 408–421. (10.1111/j.1365-2435.2007.01246.x)

[2] Badyaev AV, Uller T 2009 Parental effects in ecology and evolution: mechanisms, processes and implications. Phil. Trans. R. Soc. B 364, 1169–1177. (10.1098/rstb.2008.0302) 19324619 PMC2666689

[3] Wolf JB, Wade MJ 2009 What are maternal effects (and what are they not)? Phil. Trans. R. Soc. B 364, 1107–1115. (10.1098/rstb.2008.0238) 19324615 PMC2666680

[4] Bernardo J 1996 Maternal effects in animal ecology. Am. Zool. 36, 83–105. (10.1093/icb/36.2.83)

[5] Rossiter M 1996 Incidence and consequences of inherited environmental effects. Annu. Rev. Ecol. Syst. 27, 451–476. (10.1146/annurev.ecolsys.27.1.451)

[6] Mousseau TA, Fox CW 1998 The adaptive significance of maternal effects. Trends Ecol. Evol. 13, 403–407. (10.1016/S0169-5347(98)01472-4) 21238360

[7] Hereford J, Moriuchi K 2005 Variation among populations of diodia teres (rubiaceae) in environmental maternal effects. J. Evol. Biol. 18, 124–131. (10.1111/j.1420-9101.2004.00797.x) 15669968

[8] Beckerman AP, Benton TG, Lapsley CT, Koesters N 2006 How effective are maternal effects at having effects? Proc. R. Soc. B 273, 485–493. (10.1098/rspb.2005.3315) PMC1560202 16615217

[9] Grindstaff JL, Brodie ED, Ketterson ED 2003 Immune function across generations: integrating mechanism and evolutionary process in maternal antibody transmission. Proc. R. Soc. Lond. B 270, 2309–2319. (10.1098/rspb.2003.2485) PMC1691520 14667346

[10] Hasselquist D, Nilsson JA 2009 Maternal transfer of antibodies in vertebrates: trans-generational effects on offspring immunity. Phil. Trans. R. Soc. B 364, 51–60. (10.1098/rstb.2008.0137) 18926976 PMC2666691

[11] Little TJ, Kraaijeveld AR 2004 Ecological and evolutionary implications of immunological priming in invertebrates. Trends Ecol. Evol. 19, 58–60. (10.1016/j.tree.2003.11.011) 16701227

[12] Milutinović B, Kurtz J 2016 Immune memory in invertebrates. Semin. Immunol. 28, 328–342. (10.1016/j.smim.2016.05.004) 27402055

[13] Pigeault R, Garnier R, Rivero A, Gandon S 2016 Evolution of transgenerational immunity in invertebrates. Proc. R. Soc. B 283, 20161136 (10.1098/rspb.2016.1136) PMC5046895 27683366

[14] Hoffmann JA, Reichhart JM 2002 *Drosophila* innate immunity: an evolutionary perspective. Nat. Immunol. 3, 121–126. (10.1038/ni0202-121) 11812988

[15] Kurtz J 2005 Specific memory within innate immune systems. Trends Immunol. 26, 186–192. (10.1016/j.it.2005.02.001) 15797508

[16] Schmid-Hempel P 2005 Natural insect host–parasite systems show immune priming and specificity: puzzles to be solved. Bioessays 27, 1026–1034. (10.1002/bies.20282) 16163710

[17] Schmid-Hempel P 2005 Evolutionary ecology of insect immune defenses. Annu. Rev. Entomol. 50, 529–551. (10.1146/annurev.ento.50.071803.130420) 15471530

[18] Kurtz J, Franz K 2003 Innate defence: evidence for memory in invertebrate immunity. Nature 425, 37–38. (10.1038/425037a) 12955131

[19] Sadd BM, Schmid-Hempel P 2006 Insect immunity shows specificity in protection upon secondary pathogen exposure. Curr. Biol. 16, 1206–1210. (10.1016/j.cub.2006.04.047) 16782011

[20] Pham LN, Dionne MS, Shirasu-Hiza M, Schneider DS 2007 A specific primed immune response in *Drosophila* is dependent on phagocytes. PLoS Pathog. 3, e26 (10.1371/journal.ppat.0030026) 17352533 PMC1817657

[21] Roth O, Sadd BM, Schmid-Hempel P, Kurtz J 2009 Strain-specific priming of resistance in the red flour beetle, *Tribolium castaneum*. Proc. R. Soc. B 276, 145–151. (10.1098/rspb.2008.1157) PMC2614262 18796392

[22] Tate AT, Rudolf VH 2012 Impact of life stage specific immune priming on invertebrate disease dynamics. Oikos 121, 1083–1092. (10.1111/j.1600-0706.2011.19725.x)

[23] Watson FL, Püttmann-Holgado R, Thomas F, Lamar DL, Hughes M, Kondo M, Rebel VI, Schmucker D 2005 Extensive diversity of Ig-superfamily proteins in the immune system of insects. Science 309, 1874–1878. (10.1126/science.1116887) 16109846

[24] Kurtz J, Armitage SA 2006 Alternative adaptive immunity in invertebrates. Trends Immunol. 27, 493–496. (10.1016/j.it.2006.09.001) 16979938

[25] Little TJ, Hultmark D, Read AF 2005 Invertebrate immunity and the limits of mechanistic immunology. Nat. Immunol. 6, 651 (10.1038/ni1219) 15970937

[26] Pinaud S *et al.* 2016 A shift from cellular to humoral responses contributes to innate immune memory in the vector snail *Biomphalaria glabrata*. PLoS Pathog. 12, 1–18. (10.1371/journal.ppat.1005361) PMC4703209 26735307

[27] Barribeau SM, Schmid-Hempel P, Sadd BM 2016 Royal decree: gene expression in trans-generationally immune primed bumblebee workers mimics a primary immune response. PLoS ONE 11, e0159635 (10.1371/journal.pone.0159635) 27442590 PMC4956190

[28] Tate AT, Andolfatto P, Demuth JP, Graham AL 2017 The within-host dynamics of infection in trans-generationally primed flour beetles. Mol. Ecol. 26, 3794–3807. (10.1111/mec.14088) 28277618 PMC5653231

[29] Netea MG, Joosten LAB, Latz E, Mills KHG, Natoli G, Stunnenberg HG, O’Neill LAJ, Xavier RJ 2016 Trained immunity: a program of innate immune memory in health and disease. Science 352, aaf1098 (10.1126/science.aaf1098) 27102489 PMC5087274

[30] Ben-Ami F, Ebert D, Regoes RR 2010 Pathogen dose infectivity curves as a method to analyze the distribution of host susceptibility: a quantitative assessment of maternal effects after food stress and pathogen exposure. Am. Nat. 175, 106–115. (10.1086/648672) 19911987

[31] Boots M, Roberts KE 2012 Maternal effects in disease resistance: poor maternal environment increases offspring resistance to an insect virus. Proc. R. Soc. B 279, 4009–4014. (10.1098/rspb.2012.1073) PMC3427573 22833270

[32] Michel J, Ebert D, Hall MD 2016 The trans-generational impact of population density signals on host-parasite interactions. BMC Evol. Biol. 16, 254 (10.1186/s12862-016-0828-4) 27887563 PMC5123254

[33] Roth O, Joop G, Eggert H, Hilbert J, Daniel J, Schmid-Hempel P, Kurtz J 2010 Paternally derived immune priming for offspring in the red flour beetle, *Tribolium castaneum*. J. Anim. Ecol. 79, 403–413. (10.1111/j.1365-2656.2009.01617.x) 19840170

[34] Hernández López J, Schuehly W, Crailsheim K, Riessberger-Gallé U 2014 Trans-generational immune priming in honeybees. Proc. Biol. Sci. 281, 20140454 (10.1098/rspb.2014.0454) 24789904 PMC4024302

[35] McNamara KB, van Lieshout E, Simmons LW 2014 The effect of maternal and paternal immune challenge on offspring immunity and reproduction in a cricket. J. Evol. Biol. 27, 1020–1028. (10.1111/jeb.12376) 24750259

[36] Hall MD, Ebert D 2012 Disentangling the influence of parasite genotype, host genotype and maternal environment on different stages of bacterial infection in *Daphnia magna*. Proc. R. Soc. B 279, 3176–3183. (10.1098/rspb.2012.0509) PMC3385728 22593109

[37] Schlotz N, Ebert D, Martin-Creuzburg D 2013 Dietary supply with polyunsaturated fatty acids and resulting maternal effects influence host–parasite interactions. BMC Ecol. 13, 41 (10.1186/1472-6785-13-41) 24175981 PMC3826666

[38] Pigeault R, Vézilier J, Nicot A, Gandon S, Rivero A 2015 Transgenerational effect of infection in *Plasmodium*-infected mosquitoes. Biol. Lett. 11, 20141025 (10.1098/rsbl.2014.1025) 25762571 PMC4387496

[39] Huang CC, Song YL 1999 Maternal transmission of immunity to white spot syndrome associated virus (WSSV) in shrimp (*Penaeus monodon*). Dev. Comp. Immunol. 23, 545–552. (10.1016/S0145-305X(99)00038-5) 10579383

[40] Sadd BM, Kleinlogel Y, Schmid-Hempel R, Schmid-Hempel P 2005 Trans-generational immune priming in a social insect. Biol. Lett. 1, 386–388. (10.1098/rsbl.2005.0369) 17148213 PMC1626361

[41] Moret Y 2006 Trans-generational immune priming: specific enhancement of the antimicrobial immune response in the mealworm beetle, *Tenebrio molitor*. Proc. R. Soc. B 273, 1399–1405. (10.1098/rspb.2006.3465) PMC1560290 16777729

[42] Tidbury HJ, Pedersen AB, Boots M 2011 Within and transgenerational immune priming in an insect to a DNA virus. Proc. R. Soc. B 278, 871–876. (10.1098/rspb.2010.1517) PMC3049047 20861049

[43] Khan I, Prakash A, Agashe D 2017 Experimental evolution of insect immune memory versus pathogen resistance. Proc. R. Soc. B 284, 20171583 (10.1098/rspb.2017.1583) PMC5745399 29237849

[44] Norouzitallab P, Baruah K, Biswas P, Vanrompay D, Bossier P 2016 Probing the phenomenon of trained immunity in invertebrates during a transgenerational study, using brine shrimp *Artemia* as a model system. Sci. Rep. 6, 21166 (10.1038/srep21166) 26876951 PMC4753410

[45] Little TJ, O’Connor B, Colegrave N, Watt K, Read AF 2003 Maternal transfer of strain-specific immunity in an invertebrate. Curr. Biol. 13, 489–492. (10.1016/S0960-9822(03)00163-5) 12646131

[46] Regoes RR, Hottinger JW, Sygnarski L, Ebert D 2003 The infection rate of *Daphnia magna* by *Pasteuria ramosa* conforms with the mass-action principle. Epidemiol. Infect. 131, 957–966. (10.1017/S0950268803008793) 14596538 PMC2870041

[47] Ben-Ami F, Regoes RR, Ebert D 2008 A quantitative test of the relationship between parasite dose and infection probability across different host-parasite combinations. Proc. R. Soc. B 275, 853–859. (10.1098/rspb.2007.1544) PMC2596906 18198145

[48] Halloran ME, Longini IM Jr, Struchiner CJ 1996 Estimability and interpretation of vaccine efficacy using frailty mixing models. Am. J. Epidemiol. 144, 83–97. (10.1093/oxfordjournals.aje.a008858) 8659489

[49] Longini IM, Halloran ME 1996 A frailty mixture model for estimating vaccine efficacy. Appl. Stat. 45, 165–173. (10.2307/2986152)

[50] Halloran E, Ira M, Longini J, Struchiner C 2010 Design and analysis of vaccine studies. Statistics for Biology and Health Springer ISBN: 9780387403137. See http://books.google.ch/books?id=RIlItQAACAAJ.

[51] Furumoto WA, Mickey R 1967 A mathematical model for the infectivity-dilution curve of tobacco mosaic virus: theoretical considerations. Virology 32, 216–223. (10.1016/0042-6822(67)90271-1) 6025875

[52] Haas CN 1999 Quantitative microbial risk assessment. New York, NY: Wiley ISBN: 0-471-18397-0.

[53] Carius HJ, Little TJ, Ebert D 2001 Genetic variation in a host-parasite association: potential for coevolution and frequency-dependent selection. Evolution 55, 1136–1145. (10.1111/j.0014-3820.2001.tb00633.x) 11475049

[54] Rowley AF, Powell A 2007 Invertebrate immune systems specific, quasi-specific, or nonspecific? J. Immunol. 179, 7209–7214. (10.4049/jimmunol.179.11.7209) 18025161

[55] Hauton C, Smith VJ 2007 Adaptive immunity in invertebrates: a straw house without a mechanistic foundation. Bioessays 29, 1138–1146. (10.1002/bies.20650) 17935208

[56] Roth O, Beemelmanns A, Barribeau SM, Sadd BM 2018 Recent advances in vertebrate and invertebrate transgenerational immunity in the light of ecology and evolution. Heredity (Edinb) 121, 225–238. (10.1038/s41437-018-0101-2) 29915335 PMC6082847

[57] Ben-Ami F, Routtu J 2013 The expression and evolution of virulence in multiple infections: the role of specificity, relative virulence and relative dose. BMC Evol. Biol. 13, 97 (10.1186/1471-2148-13-97) 23641899 PMC3659053

[58] Mitchell SE, Read AF 2005 Poor maternal environment enhances offspring disease resistance in an invertebrate. Proc. R. Soc. B 272, 2601–2607. (10.1098/rspb.2005.3253) PMC1559984 16321782

[59] Bandilla M, Hakalahti T, Hudson PJ, Valtonen ET 2005 Aggregation of *Argulus coregoni* (Crustacea: Branchiura) on rainbow trout (*Oncorhynchus mykiss*): a consequence of host susceptibility or exposure? Parasitology 130, 169–176. (10.1017/S0031182004006407) 15727066

[60] Wang S, Spear RC 2016 Exposure versus susceptibility as alternative bases for new approaches to surveillance for *Schistosoma japonicum* in low transmission environments. PLoS Negl. Trop. Dis. 10, e0004425 (10.1371/journal.pntd.0004425) 26942912 PMC4778868

[61] Brites D, McTaggart S, Morris K, Anderson J, Thomas K, Colson I, Fabbro T, Little TJ, Ebert D, Du Pasquier L 2008 The Dscam homologue of the crustacean *Daphnia* is diversified by alternative splicing like in insects. Mol. Biol. Evol. 25, 1429–1439. (10.1093/molbev/msn087) 18403399

[62] Garbutt JS, Little TJ 2017 Bigger is better: changes in body size explain a maternal effect of food on offspring disease resistance. Ecol Evol 7, 1403–1409. (10.1002/ece3.2709) 28261452 PMC5330872

[63] Luijckx P, Ben-Ami F, Mouton L, Du Pasquier L, Ebert D 2011 Cloning of the unculturable parasite *Pasteuria ramosa* and its *Daphnia* host reveals extreme genotype-genotype interactions. Ecol. Lett. 14, 125–131. (10.1111/j.1461-0248.2010.01561.x) 21091597

[64] Ben-Ami F 2019 Host age effects in invertebrates: epidemiological, ecological, and evolutionary implications. Trends Parasitol. 35, 466–480. (10.1016/j.pt.2019.03.008) 31003758

[65] Graw F, Magnus C, Regoes RR 2010 Theoretical analysis of the evolution of immune memory. BMC Evol. Biol. 10, 380 (10.1186/1471-2148-10-380) 21143840 PMC3018457

[66] Mayer A, Mora T, Rivoire O, Walczak AM 2016 Diversity of immune strategies explained by adaptation to pathogen statistics. Proc. Natl Acad. Sci. USA 113, 8630–8635. (10.1073/pnas.1600663113) 27432970 PMC4978245

[67] Struchiner CJ, Halloran ME, Robins JM, Spielman A 1990 The behaviour of common measures of association used to assess a vaccination programme under complex disease transmission patterns–a computer simulation study of malaria vaccines. Int. J. Epidemiol. 19, 187–196. (10.1093/ije/19.1.187) 2351514

[68] Halloran ME, Haber M, Longini IM Jr 1992 Interpretation and estimation of vaccine efficacy under heterogeneity. Am. J. Epidemiol. 136, 328–343. (10.1093/oxfordjournals.aje.a116498) 1415152

[69] Hudgens MG, Gilbert PB 2009 Assessing vaccine effects in repeated low-dose challenge experiments. Biometrics 65, 1223–1232. (10.1111/j.1541-0420.2009.01189.x) 19397589 PMC2794923

[70] Langwig KE *et al.* 2017 Vaccine effects on heterogeneity in susceptibility and implications for population health management. MBio 8, e00796–17. (10.1128/mBio.00796-17) 29162706 PMC5698548

[71] Regoes RR 2012 The role of exposure history on HIV acquisition: insights from repeated low-dose challenge studies. PLoS Comput. Biol. 8, e1002767 (10.1371/journal.pcbi.1002767) 23180981 PMC3493490

[72] Green J 1974 Parasites and epibionts of cladocera. J. Zool. 32, 417–515.

[73] Ebert D 2005 Ecology, epidemiology, and evolution of parasitism in Daphnia. Bethesda, MD; National Library of Medicine.

[74] Goren L, Ben-Ami F 2013 Ecological correlates between cladocerans and their endoparasites from permanent and rain pools: patterns in community composition and diversity. Hydrobiologia 701, 13–23. (10.1007/s10750-012-1243-5)

[75] Ebert D, Rainey P, Embley TM, Scholz D 1996 Development, life cycle, ultrastructure and phylogenetic position of *Pasteuria ramosa* Metchnikoff 1888: rediscovery of an obligate endoparasite of *Daphnia magna* Straus. Phil. Trans. R. Soc. Lond. B 351, 1689–1701. (10.1098/rstb.1996.0151)

[76] Ebert D, Duneau D, Hall MD, Luijckx P, Andras JP, Du Pasquier L, Ben-Ami F 2016 A population biology perspective on the stepwise infection process of the bacterial pathogen *Pasteuria ramosa* in *Daphnia*. In Advances in parasitology, vol. 91, pp. 265–310. Elsevier. 27015951 10.1016/bs.apar.2015.10.001

[77] Mageroy JH, Grepperud EJ, Jensen KH 2011 Who benefits from reduced reproduction in parasitized hosts? An experimental test using the *Pasteuria ramosa-Daphnia magna* system. Parasitology 138, 1910–1915. (10.1017/S0031182011001302) 21854675

[78] Clerc M, Ebert D, Hall MD 2015 Expression of parasite genetic variation changes over the course of infection: implications of within-host dynamics for the evolution of virulence. Proc. R. Soc. B 282, 20142820 (10.1098/rspb.2014.2820) PMC4375866 25761710

[79] Little TJ, Watt K, Ebert D 2006 Parasite-host specificity: experimental studies on the basis of parasite adaptation. Evolution 60, 31–38. (10.1111/j.0014-3820.2006.tb01079.x) 16568629

[80] Ebert D, Zschokke-Rohringer CD, Carius HJ 1998 Within- and between-population variation for resistance of *Daphnia magna* to the bacterial endoparasite *Pasteuria ramosa*. Proc. R. Soc. B: Biol. Sci. 265, 2127–2134. (10.1098/rspb.1998.0549)

