# Supplementary material for: Disentangling non-specific and specific transgenerational immune priming components in host–parasite interactions
Source: Proc Biol Sci. 2020 Feb 12;287(1920):20192386. doi: 10.1098/rspb.2019.2386 (PMC7031663; doi:10.1098/rspb.2019.2386)
Supplement: Supplementary Text and Figures [file rspb20192386supp1.pdf]

Electronic Supplementary Material

S.1 Mathematical modeling

Here, we first briefly describe the *heterogeneous susceptibility model* developed and used in Regoes et al. (2003), Ben-Ami et al. (2008, 2010). We then describe how we extended this model to investigate the potential non-specific and specific immune priming effects of maternal exposure.

S.1.1 The heterogeneous susceptibility model

The heterogeneous susceptibility model describes a host population in which individuals differ in their susceptibility to infection with a pathogen. It is based on frailty mixing models commonly used in epidemiology (Halloran et al., 1996, Longini and Halloran, 1996, Halloran et al., 2010), and can be used to infer susceptibility distributions from multi-dose challenge data (Regoes et al., 2003, Ben-Ami et al., 2008, 2010).

For the sake of completeness, we reiterate the definition of the heterogeneous susceptibility model here. Let us first ignore that we used different parasite isolates in our experiment, and assume that we challenge *Daphnia* hosts with a generic strain, and that the mothers of these *Daphnia* were not exposed to any parasite. Let P be the dose of this strain, measured in spores per milliliter medium. The heterogeneous susceptibility model as described in Ben-Ami et al. (2008, 2010), assumes that the infection hazard, λ, is proportional to the parasite dose to which the the *Daphnia* are exposed:

$$\lambda = b\ P \tag{1}$$

The proportionality factor b is the infection rate constant, and comprises the susceptibility of the host as well as the infectivity of the parasite. Under the assumption that parasite spores, P, are not destroyed or cleared during the exposure duration t_e, the cumulative hazard, Λ, is given by:

$$\Lambda = \int_0^{t_e} b\ P\ dt = b\ P\ t_e \tag{2}$$

In the heterogeneous susceptibility model, we assume that the susceptibility to the parasite varies across host individuals. Formally, we implement this into the model by assuming that the infection rate constant, b, is drawn from a Γ-distribution for each host individual. Let $D_{\bar{b},\,\nu}(b)$ denote the density of a Γ-distribution with mean $\bar{b}$ and variance parameter ν. Mathematically, the variance parameter ν is the inverse of the shape parameter of the Γ-distribution. We can then write the proportion of hosts that remain uninfected after the time t_e as:

$$S = \int_0^{\infty} e^{-b\ P\ t_e}\ D_{\bar{b},\,\nu}(b)\ db \tag{3}$$

$$= \left(\frac{1}{1+\bar{b}\ P\ t_e\ \nu}\right)^{\frac{1}{\nu}} \tag{4}$$

From this equation, we can derive an expression for the ID_{50} by setting $S = 1/2$:

$$\mathrm{ID}_{50} = \frac{2^{\nu} - 1}{\bar{b}\, t_e\, \nu} \tag{5}$$

From the expression of S in Equation 4, we can construct a likelihood. In our experiments, we exposed *Daphnia* to different parasite doses. Let us index these doses by d, meaning that $d = 1$ denotes the first dose we applied in our experiment. The corresponding doses are denoted by P_d. Further, let n_d denote the number of *Daphnia* exposed to the dth dose, of which i_d become infected. The likelihood L_d of data at the dth dose level can then be written as:

$$L_d(\bar{b}, \nu | n_d, i_d) = S_d^{n_d - i_d}\,(1 - S_d)^{i_d} \tag{6}$$

where

$$S_d = \left(\frac{1}{1 + \bar{b}\, P_d\, t_e\, \nu}\right)^{\frac{1}{\nu}} \tag{7}$$

Our experiments involve exposures to seven doses, that is $d = 1, \ldots, 7$. Let us denote the full data-set by $\boldsymbol{n} = (n_1, \ldots, n_7)$ and $\boldsymbol{i} = (i_1, \ldots, i_7)$. The likelihood of the full data-set is:

$$L(\bar{b}, \nu | \boldsymbol{n}, \boldsymbol{i}) = \prod_{d=1}^{7} L_d(\bar{b}, \nu | n_d, i_d) \tag{8}$$

S.1.2 Including non-specific and specific immune priming effects

Let us now incorporate into this framework the potential non-specific and specific immune priming effects of maternal parasite exposure.

Our experiments involve three isolates of *Pasteuria ramosa*, P1, P2, P5. These isolates are used in two different ways. First, they are used to challenge the mother generation. This results in four treatment groups of offspring, three with maternal exposure to either P1, P2, or P5, respectively, and a control group of *Daphnia* the mothers of which were not exposed. Second, the three strains are used to challenge the offspring. Challenging *Daphnia* from each of the four groups with each of the three *P. ramosa* isolates thus leads to twelve groups.

In each of these groups, the susceptibility to the parasite, $\bar{b}$, and its variance, ν, will differ. We therefore index the susceptibility and variance parameter by the maternal ($i = 0, 1, 2, 5$) and offspring parasite isolate ($j = 1, 2, 5$), resulting in two sets of parameters $\bar{b}_{ij}$, and ν_{ij}. Hereby, the the values 1,2, and 5 of the indices i and j correspond to challenge of mothers or offspring with P1, P2, and P5, respectively, and $i = 0$ corresponds to the control group, the mothers of which have not been exposed to any parasite isolate. For example, the parameter $\bar{b}_{01}$ denotes the average susceptibility of the offspring population, the mothers of which were not exposed to the parasite and that were challenged with P1, and ν_{55} denotes the variance parameter of the susceptibility distribution of *Daphnia*, the mothers of which were exposed to P5, and that were also challenged with P5.

Comparisons between the parameters characterizing the twelve groups allow us to infer non-specific or specific immune priming effects. For example, non-specific immune priming by isolate P1 can be measured by comparing the mean of $\bar{b}_{1j}$, to the

mean of $\bar{b}_{0j}$, $j = 1, 2, 3$. Specific immune priming by isolate P1 can be measured by comparing $\bar{b}_{11}$ to the mean of $\bar{b}_{21}$ and $\bar{b}_{51}$. This difference tells us if there is specific memory of P1. However, such comparisons are cumbersome with this treatment-group-centered parameterization.

To be able to disentangle non-specific from specific immune priming effects in a more straightforward fashion, we provide an alternative parameterization for these two sets of parameters, $\bar{b}_{ij}$ and ν_{ij}, $i = 0, 1, 2, 5$ and $j = 1, 2, 5$. We re-parameterized the average susceptibility parameters in the following way:

$$b_{ij} = b_{0j}(1 - r_i)(1 - \delta_{ij} m_j) \tag{9}$$

On the right-hand side of Equation 9, the mean susceptibility of this population can be decomposed into three terms. The first is the baseline susceptibility, b_{0j}, of *Daphnia* to Pj. The second term, $1 - r_i$, describes the potential reduction of the baseline susceptibility due to exposure of the mothers to Pi. The value of the reduction parameter, r_i should be $0 \leq r_i \leq 1$, although $r_i < 0$ is also conceivable and would correspond to an increase due to exposure of the mothers. This term measures non-specific immune priming — non-specific because it does not matter here if $i = j$ or $i \neq j$, that is, if the challenge is homologous or heterologous. The third term, $1 - \delta_{ij} m_j$, describes specific immune priming or memory, that is a potential further reduction of susceptibility if the mothers have been exposed to the same strain with which the offspring are challenges, Pj. The parameter δ_{ij} denotes Kronecker's delta, which is 1 for $i = j$, and 0 otherwise. The value of the specific immunity parameter, m_j should be $0 \leq m_j \leq 1$, although $m_j < 0$ is also conceivable and would correspond to a specific increase of susceptibility (the opposite of immune memory).

For our 3×3 treatment groups, b_{ij} in Equation 9 can be written in matrix form:

$$\begin{pmatrix} b_{11} & b_{12} & b_{15} \\ b_{21} & b_{22} & b_{25} \\ b_{51} & b_{52} & b_{55} \end{pmatrix} = \begin{pmatrix} b_{01}(1 - r_1)(1 - m_1) & b_{02}(1 - r_1) & b_{05}(1 - r_1) \\ b_{01}(1 - r_2) & b_{02}(1 - r_2)(1 - m_2) & b_{05}(1 - r_2) \\ b_{01}(1 - r_5) & b_{02}(1 - r_5) & b_{05}(1 - r_5)(1 - m_5) \end{pmatrix} \tag{10}$$

In this matrix form, the contribution of each of the three components — baseline susceptibility b_{0j}, non-specific susceptibility reduction r_i and specific immune priming m_i — is easier to grasp than in Equation 9. Figure S1 visualizes this susceptibility matrix, showing how the susceptibility of each of the 3×3 treatments is built up from baseline susceptibility, non-specific susceptibility reduction and specific immune priming.

In addition to the mean susceptibility parameters b_{ij}, the extended heterogeneous susceptibility model features the variance parameters ν_{ij}. To capture baseline variances, as well as non-specific and specific effects of maternal parasite exposure, we re-parameterize these variance parameters analogously to the mean susceptibility parameters:

$$\nu_{ij} = \nu_{0j}(1 - \rho_i)(1 - \delta_{ij} \mu_j) \tag{11}$$

Hereby, ν_{0j} denotes the baseline variance of the susceptibility distribution of control *Daphnia* to Pj. $1 - \rho_i$, describes the potential reduction of the baseline variance due

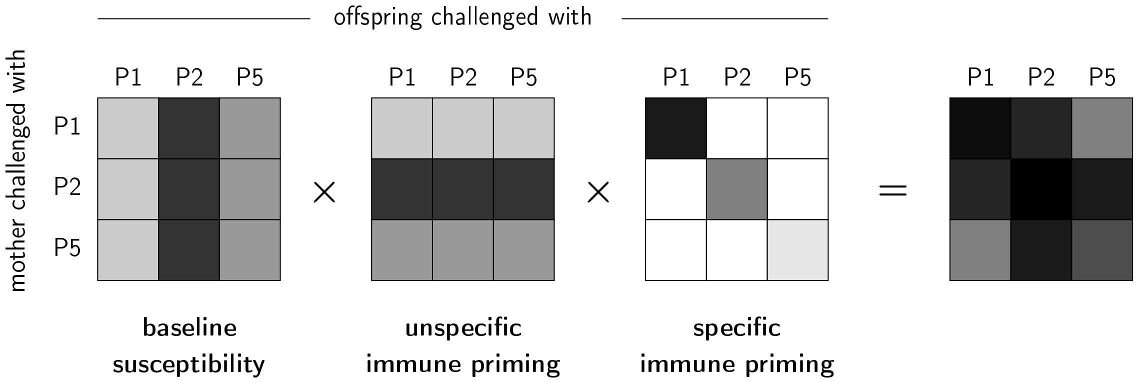

Figure S1: Decomposing the susceptibilities into heterologous and homologous components. The matrix on the left hand side represents the susceptibilities of the offspring of the control group to each parasite strain. The lighter the color, the higher the susceptibility. The second left matrix illustrates non-specific effects of priming with each parasite strain on offspring susceptibility. White colored entries signify no effect, darker entries signify reductions in susceptibilities. The third matrix from the left captures a potential memory effect. Again dark signifies a strong specific immunization effect. All of these components are synthesized to the matrix on the right by "visually multiplying" the shades of gray entry by entry.

to exposure of the mothers to Pi. Lastly, $1-\delta_{ij}\mu_j$, describes an effect on the variance of the susceptibility distribution that applies to homologous challenges only.

S.2 Model selection and fitting

This parameterization of the heterogeneous susceptibility model gives rise to many model variants. These are listed and described in Table 1. We used a model selection scheme shown in Figure 3 to determine which model variant is most consistent with the experimental data.

Models with varying degrees of complexity were fitted to the data, and the fits were compared with a likelihood ratio test. Parameters were estimated by maximizing the likelihood of the fitted model.

S.3 Implementation

All the mathematical models and the analysis described in the previous sections were implemented in the R language of statistical computing (R Core Team, 2013). Likelihood functions were maximized using `optim()` and the `mle2()` in the package `bbmle` (Bolker and Team, 2017). Confidence intervals were estimated using the function `confint.mle2()` on the mle2-class fitting objects with the default spline-extrapolation-based method. The experimental data and the essential R-functions needed to recapitulate our analysis are provided in the electronic supplementary file `Ben-Ami-ESM-Data.csv` and `Ben-Ami-ESM-Code.R`, respectively.

Standard error for the ID_{50} estimates were derived by a parametric bootstrap routine. Specifically, we resampled our infection data for each dose level in each

treatment group by drawing a random numbers of infected host individuals from a binomial distribution. We set the size parameter of this binomial distribution equal to the number of host individuals that were exposed to a given pathogen dose in a given group in our experiments, and set the success probability of the binomial to the fraction of infected hosts for this dose in this group. For 1000 resampled datasets, we then estimated the parameters of the best supported $r - m_i$ model and calculated the ID_{50} from Equation 5.

S.4 The relationship of our inference framework to generalized linear models

The most common framework applied to analyze infection experiments are generalized linear models (McCullagh and Nelder, 1989). Here we compare our modeling and inference framework with the generalized linear modeling approach in detail. In brief, while generalized linear models can be used to address some of the questions we address with our study, their application requires careful setup of the statistical models and does not lead to conclusions that have an immediate biological interpretation.

First, the default setup of generalized linear models misrepresents several central aspects of the biological system. In particular, when the exposure dose is one of the explanatory variables, we need to root the model such that the probability of infection is zero at a zero dose, as there cannot be an infection in that case. However, in a logistic regression, an intercept at zero corresponds to an infection probability of 0.5. Correcting for this non-biological root complicates the analysis. Additional covariates, such as the strain with which the mother or the offspring generation have been challenged, should be nested within the term for the challenge dose, again to avoid the unbiological scenario of infection with zero challenge doses. Moreover, the mother parasite strain should be assumed to interact (in the statistical sense of the word) with the offspring parasite strain because it modulates the susceptibility to challenge. Thus, mapping the biology correctly to a generalized linear modeling framework requires three-way interaction terms with the appropriate nesting of factors.

Second, the parameterization of our modeling framework allows an immediate biological interpretation of the estimated parameters. In the context of generalized linear models, in contrast, it will be difficult to derive the baseline offspring susceptibility, its reduction due to maternal exposure, and a memory effect from the estimated three-way interaction terms. Most importantly, the distinction between homologous and heterologous challenge cannot be implemented in a generalized linear model without defining an additional factor describing the nature of the challenge that depends on the parasite strain with which the mother and offspring have been challenged. Note that disentangling differences between homologous and heterologous challenges requires a more specific parameterization than the "interaction terms" between mother and offspring parasite strains in a generalized linear model. Isolating the differential effect of homologous and heterologous challenges requires parameters $(1 - m_j)$ that scale the diagonal entries. Interaction terms, in contrast, allow simply twelve different infection rate constants for each mother and offspring

parasite strain.

Lastly, generalized linear models using the binomial error distribution essentially assume homogeneous susceptibilities, and thus do not allow to account for and estimate the variance in susceptibility across hosts. Our approach, in contrast, has its roots in our earlier studies aiming to specifically quantify the levels of heterogeneity in susceptibility by adopting frailty models from statistics and mathematical epidemiology. Heterogeneity in susceptibility is captured in the variance parameters ν_i, the estimation of which requires challenges with a range of doses.

References

Ben-Ami F, Ebert D & Regoes RR (2010). **Pathogen dose infectivity curves as a method to analyze the distribution of host susceptibility: a quantitative assessment of maternal effects after food stress and pathogen exposure**. *Am Nat* **175**(1):106–115.

Ben-Ami F, Regoes RR & Ebert D (2008). **A quantitative test of the relationship between parasite dose and infection probability across different host-parasite combinations**. *Proc Biol Sci* **275**(1636):853–859.

Bolker B & Team RDC. **bbmle: Tools for General Maximum Likelihood Estimation**, 2017.

Halloran E, Ira M. Longini J & Struchiner C. **Design and Analysis of Vaccine Studies**. Statistics for Biology and Health. Springer, 2010. ISBN 9780387403137.

Halloran ME, Longini IM Jr & Struchiner CJ (1996). **Estimability and interpretation of vaccine efficacy using frailty mixing models.** *Am J Epidemiol* **144**(1):83–97.

Longini IM & Halloran ME (1996). **A frailty mixture model for estimating vaccine efficacy**. *Appl. Statist.* **45**(2):165–173.

McCullagh P & Nelder J. **Generalized Linear Models, Second Edition**. Chapman & Hall/CRC Monographs on Statistics & Applied Probability. Taylor & Francis, 1989. ISBN 9780412317606.

R Core Team . **R: A Language and Environment for Statistical Computing**. R Foundation for Statistical Computing, Vienna, Austria, 2013.

Regoes RR, Hottinger JW, Sygnarski L & Ebert D (2003). **The infection rate of *daphnia magna* by *pasteuria ramosa* conforms with the mass-action principle**. *Epidemiology and Infection* **131**(2):957–966.

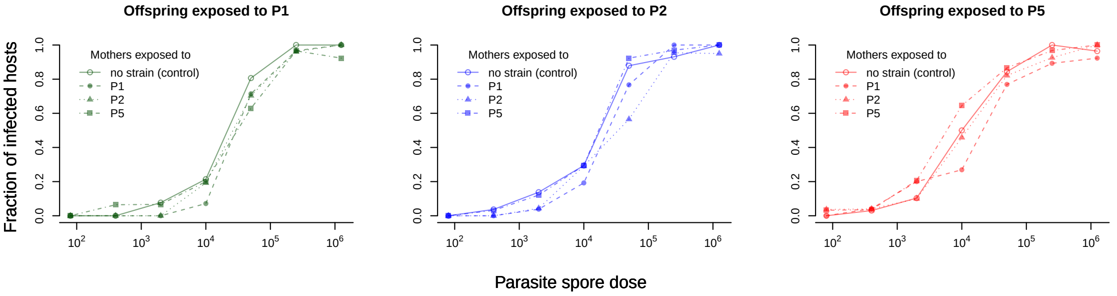

Figure S2: Fraction of infected hosts versus parasite challenge dose for each offspring treatment group. The color and lines type scheme is chosen in concordance with the experimental design schematic shown in Figure 1 and Figure 2. For a figure showing these data by maternal parasite see Figure 2.

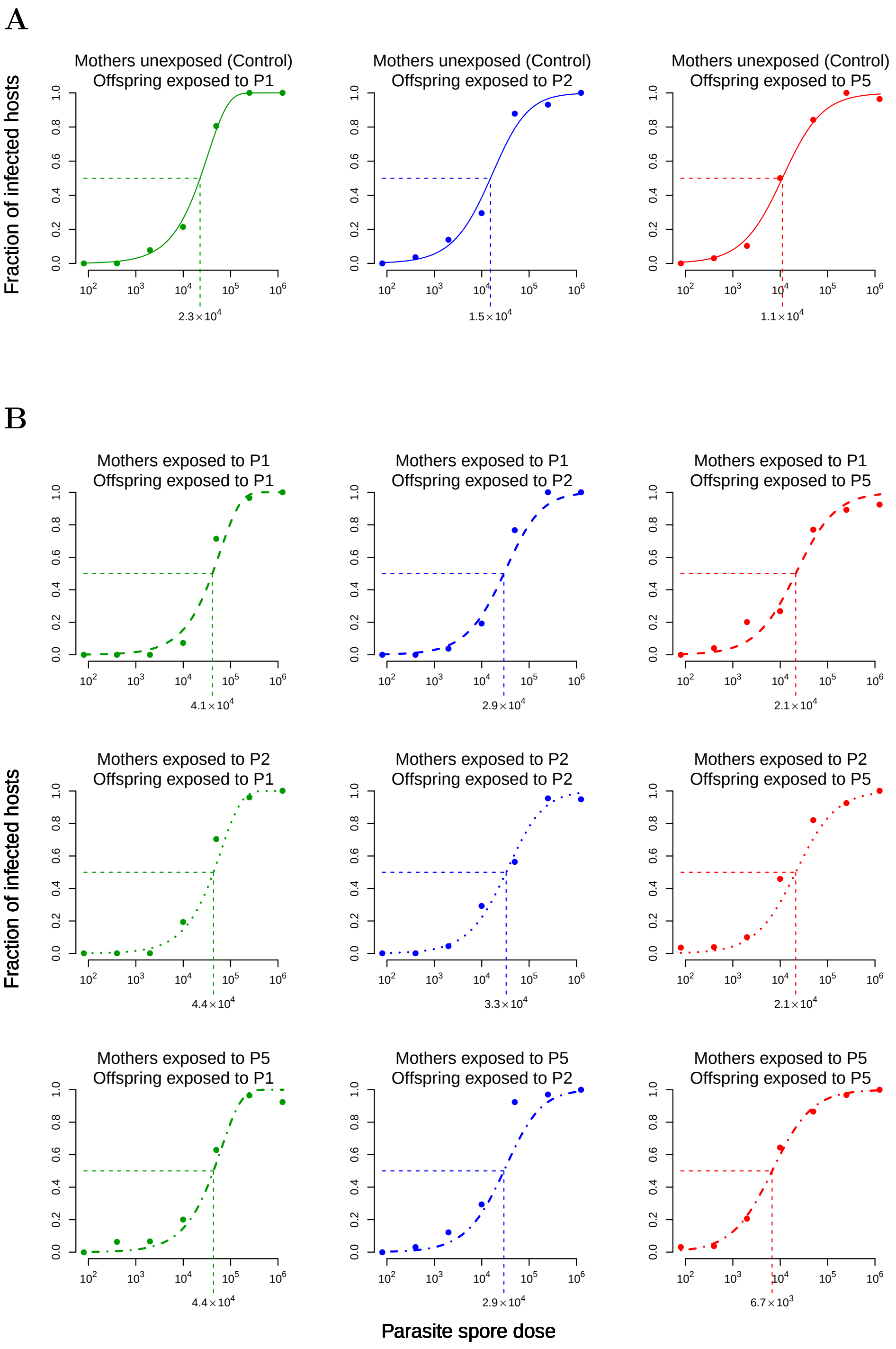

Figure S3: Model fits. (A) Fits of the heterogeneous susceptibility model to the control data. (B) Fits of the best model (the r-m_i model). The number below the x-axes gives the spore dose, at which 50% of the hosts are infected (ID_{50}). The color and lines type scheme is chosen in concordance with the experimental design schematic shown in Figure 1.

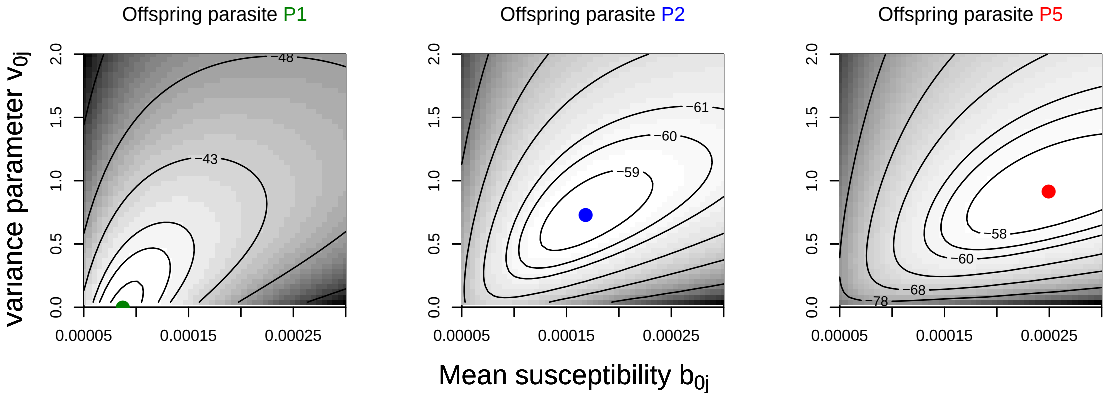

Figure S4: Likelihood surfaces for control data. This contour plots show the likelihood as the function of the model parameters β_{0j} (mean susceptibility) and ν_{0j} (variation in susceptibility) for the control groups challenged with P1, P2, and P5. The optimal parameters are marked by dots. The surfaces clearly show that there is a single maximum for each control group.
